# Biocontrol of Mycotoxigenic Fungi Using Bacteria Isolated from Ecological Vineyard Soils

**DOI:** 10.3390/jof8111136

**Published:** 2022-10-27

**Authors:** Paula de la Huerta-Bengoechea, Jéssica Gil-Serna, Clara Melguizo, Antonio J. Ramos, Montserrat Prim, Covadonga Vázquez, Belén Patiño

**Affiliations:** 1 Department of Genetics, Physiology and Microbiology, Faculty of Biology, University Complutense of Madrid, Jose Antonio Novais 12, 28040 Madrid, Spain; 2 Agrotecnio-Cerca Center, Food Technology Department, University of Lleida, Rovira Roure 191, 25198 Lleida, Spain

**Keywords:** mycotoxins, biocontrol agents, *Aspergillus*, actinobacteria, vineyards

## Abstract

The presence of mycotoxin-producing *Aspergillus* species in vineyards is a problem for food safety and the economy. In addition, rising temperatures due to climate change are modifying microbial communities, causing the replacement of some fungal species and the rise of mycotoxins such as aflatoxins. The use of microorganisms as biological control agents (BCAs) is one of the most promising strategies to prevent fungal growth and toxin production. In this study, 513 microorganisms were isolated from organic vineyard soils in different regions of Spain. The 480 bacteria and 33 yeasts isolated were sequentially screened to select those with the most suitable characteristics to be used as BCAs. After identifying 16 isolates meeting all requirements, six bacterial isolates were selected to test their potential to control three relevant toxigenic grape fungi in vitro: *A. carbonarius*, *A. niger* and *A. flavus*. Isolates of *Arthrobacter* sp., *Rhodococcus* sp. and *Bacillus mycoides* showed an excellent ability to reduce the growth and mycotoxin concentration of the above-mentioned fungi and represent potential candidates for further study regarding their possible industrial application as a BCA.

## 1. Introduction

Mycotoxins are secondary fungal metabolites that are classified as biological hazards owing to their damaging effects on human and animal health [1]. They are widely distributed across a variety of agricultural crops and pose a major risk to food safety and quality [2]. According to Eskola et al. [3], 60–80% of global crops could be contaminated with mycotoxins.

Grapes and their derivatives are highly susceptible to fungal diseases and contamination by toxin-producing fungi [4,5]. The occurrence of fungi in grapes can lead to mycotoxin contamination in both the fruit and its byproducts such as wine and juice, and this is of great concern to the economy of many countries [6]. Several fungi of the *Aspergillus*, *Alternaria* and *Penicillium* genera frequently occur in grapes, and are capable of producing mycotoxins such as ochratoxin A (OTA), aflatoxin B_1_ (AFB_1_), fumonisin B_2_ (FB_2_), alternariol, patulin, or citrinin [7]. Among these, the *Aspergillus* species are considered the most relevant, mainly *A. carbonarius* and *A. niger*, which thrive in the high temperatures of southern Europe and throughout the Mediterranean area [7,8]. The most widespread mycotoxin in grape crops is OTA [8], which is classified as a possible human carcinogen (group 2B) by the International Agency for Research on Cancer (IARC), and has immunosuppressive, teratogenic, hepatotoxic, and nephrotoxic properties [9]. In addition to OTA, the presence of aflatoxins is increasing [10,11], and of these, AFB_1_ is the most potent naturally occurring carcinogen in existence, according to the IARC, and is associated with the development of liver tumors [12].

Recent observations showed that climate change may result in the modification of existing microbial communities, which may lead to the displacement of native microbiota by microorganisms better suited to the new climatic conditions [13]. In the next 10 to 25 years, atmospheric CO_2_ concentrations are expected to double or triple, and the global temperature is expected to rise by 2 to 5 °C, which will be complemented by episodes of extreme drought [14]. These expected changes can lead to a greater dominance of aflatoxin-producing *Aspergillus* species [15]; in fact, aflatoxin contamination in vineyards has drastically increased in the last few years [10], especially where rising temperatures might favor the growth of toxigenic fungi, mainly *A. flavus* [10]. In some regions, *A. flavus* represents the third most frequent fungus isolated from grapes [10,11].

Traditional agricultural practices entail the use of chemical fungicides on crops; however, these may cause water and soil contamination, loss of crop productivity, and increased crop susceptibility to contamination by soil fungi. They also alter the physical–chemical soil balance by affecting the microorganisms responsible for maintaining its structure and decomposing organic matter [16]. Furthermore, some fungicides allowed for fungal control in vineyards are known to promote OTA production [17]. On the other hand, there is a growing public demand for a safer and more environmentally friendly alternative to control these organisms. The European Union is a major driver for the development of more integrated sustainable approaches to control pathogens and mycotoxins. Biological control stands out as one of the best responses to toxin-producing fungi although only a few commercially available products are registered [18]. In addition, it is an accepted method in organic farming, a practice which is increasing worldwide, and thus bears a wider application range. 

Biological control, or biocontrol, describes the controlled use of natural enemies of the targeted pathogens to reduce crop damage. This is mainly accomplished by adding one or more antagonistic organisms or biocontrol agents (BCAs) such as bacteria and fungi [19]. Almost any location is a potential source for new BCAs, but the preferred niche for the study and selection of these organisms is in the healthy food products, orchards, and untreated fields or organic crops. In addition, if these places have a history of contamination by toxigenic fungi, selection success is enhanced by the natural selection of their competing organisms [19]. The soil microbial community represents the largest reservoir of biological diversity in the world and may therefore be a perfect medium for finding microbial antagonists. Moreover, it is well known that soil presents the main source of toxigenic fungi in vineyards, and their spores are dispersed by wind, dust, and insects [20].

Taking all these aspects into consideration, the aim of this work was to isolate soil microorganisms from organic vineyards that could be used as potential biocontrol agents against the aforementioned toxin-producing fungi. For this purpose, the following objectives were set: (1) the sampling and isolation of microorganisms from organic soil vineyards, (2) the sequential selection and identification of isolates that meet the criteria for an ideal BCA, and (3) testing the efficacy of the selected microorganisms to control toxin-producing fungi frequently found in grapes.

## 2. Results

### 2.1. Sequential Selection of Soil-Isolated Microorganisms as Biological Control Agents

In this work, 513 microorganisms were isolated, including yeasts and bacteria. After an initial screening for lack of growth at 37 °C, only 100 were selected: 94 bacteria, 78 of which were grown on actinobacterial medium, and 6 yeasts.

The absence of growth at 37 °C is an essential criterion for avoiding the selection of potential pathogens; therefore, this condition was confirmed by incubating the isolates at this temperature over more days. After this period, 66 microorganisms were able to grow and were excluded in subsequent screenings.

The remaining 34 microorganisms (30 bacteria, 28 growing in an actinobacterial medium, and 4 yeasts) were screened according to their ability to grow under different conditions (pH, temperature and a_w_) that affect the survival of microorganisms in the soil. The results of the microorganisms’ growth under the tested conditions are shown in Table 1. Only 13 of the 28 starting actinobacteria, one bacterium and 4 yeasts were selected for identification.

The isolates AB 6-F9, AB 8-E10 and AB 8-F7 did not survive storage in glycerol so they were not identified. Polymerase chain reaction (PCR) amplification and consequent sequencing of informative regions resulted in the identification of four isolates of the yeast *Cryptococcus* sp. (Y 6-6, Y 6-7, Y 9-9 and Y 5-3), one isolate of the bacterium *Bacillus mycoides* (BC 7-B7) and four different species of actinobacteria: *Mycetocola* sp. (isolate AB 3-C10), *Pseudoarthrobacter* sp. (AB 5-D6), *Arthrobacter* sp. (AB 6-F11, AB 7-B8, AB 7-B10, AB 7-C6, AB 7-C10, AB 7-D8, and AB 10-G7), and *Rhodococcus* sp. (AB 8-G8).

All the identified yeasts were discarded for safety reasons since they belong to the genus *Cryptococcus* and some species are human pathogens. Considering they can be clones of the same individual, only one isolate of *Arthrobacter* sp. obtained from the same sample (AB 7-B8) was selected for subsequent analysis.

### 2.2. Biocontrol Assay Using the Selected Microorganisms against Toxin-Producing Fungi

**Figure 1:**
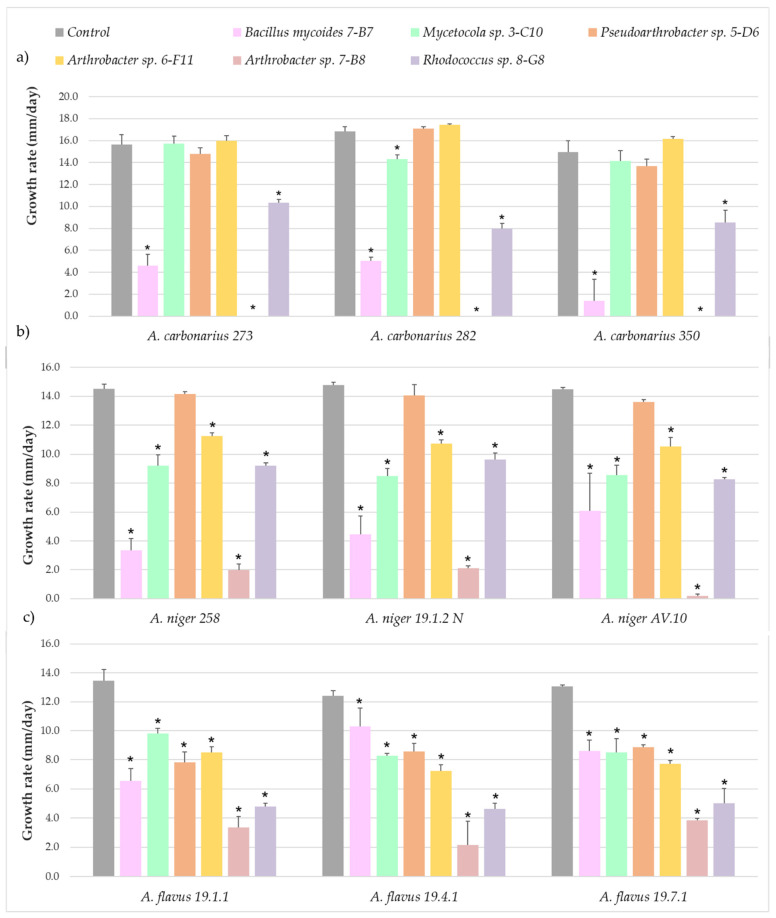
Growth rate of the three strains of (**a**) *Aspergillus carbonarius,* (**b**), *A. niger* and (**c**) *A. flavus* after being co-cultured with the corresponding bacteria. The bars indicate the mean of the three biological replicates and the thin lines their standard deviation values. The asterisks above each bar indicate statistically significant differences with respect to the control generated in Fisher’s LSD test (*p* < 0.05).

The effect of the presence of 6 selected bacterial strains in the CYA medium on the growth of *A. carbonarius*, *A. niger* and *A. flavus* is shown in Figure 1.

Three of the potential BCAs significantly reduced the fungal growth rate of the three *A. carbonarius* isolates (Figure 1a). The actinobacterium *Arthrobacter* sp. isolate 7-B8 and the bacterium *Bacillus mycoides* 7-B7 showed the greatest ability to reduce the fungal growth rate, reaching reduction percentages of up to 100% in the case of *Arthrobacter* sp. The actinobacterium *Rhodococcus* sp. (8-G8) also significantly reduced growth in the case of the three strains of *A. carbonarius*. However, the co-culture with *Mycetocola* sp. (3-C10), *Pseudoarthrobacter* sp. (5-D6), and *Arthrobacter* sp. (6-F11) did not affect fungal growth rate.

On the other hand, all but one potential BCA significantly reduced the growth of the three *A. niger* isolates (Figure 1b). Two isolates classified as *Arthrobacter* sp. (7-B8) and *Bacillus mycoides* (7-B7) showed the greatest ability to reduce fungal growth, achieving almost complete inhibition of one *A. niger* strain in the case of *Arthrobacter* sp.

All the potential BCAs significantly reduced fungal growth from the three *A. flavus* strains (Figure 1c). Two actinobacteria of the genera *Arthrobacter* (7-B8) and *Rhodococcus* (8-G8) showed the greatest ability to reduce fungal growth, with statistically significant reductions in the three fungal strains of up to 75 and 62%, respectively.

### 2.3. Quantification of Mycotoxins

**Figure 2:**
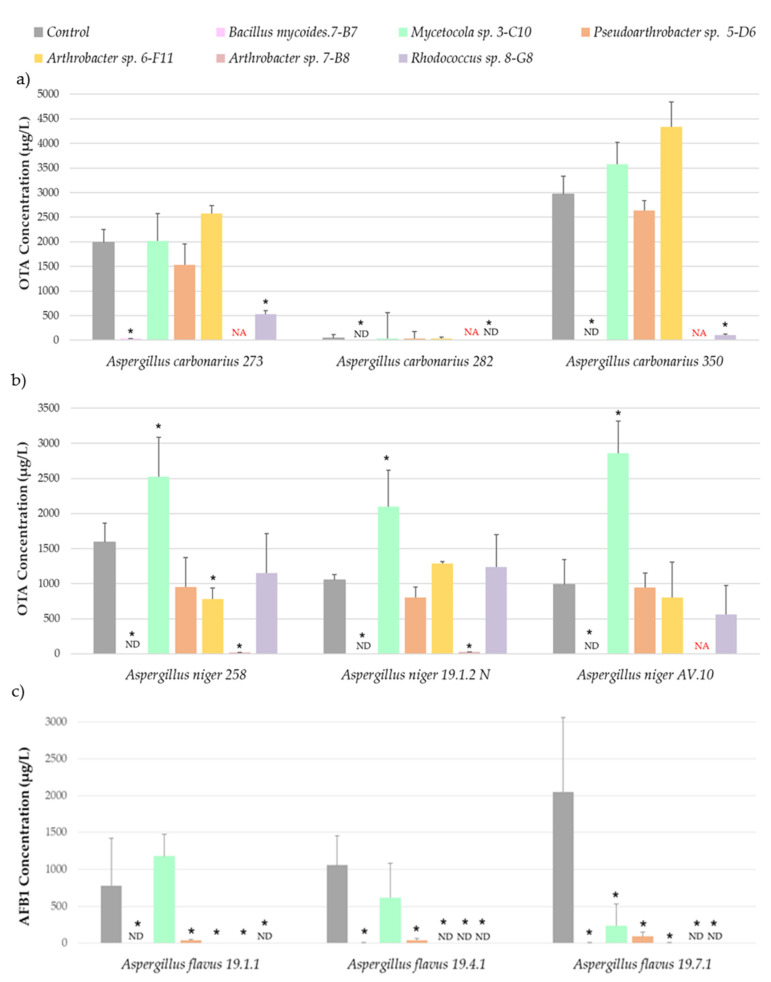
Ochratoxin A and aflatoxin B_1_ concentration of the three *Aspergillus carbonarius* (**a**), *A. niger* (**b**) and *A. flavus* (**c**) isolates tested after being co-cultured with the corresponding bacteria. The bars indicate the mean of the three biological replicates and the thin lines their standard deviation values. The asterisks above each of the bars indicate statistically significant differences with respect to the control generated in Fisher’s LSD test (*p* < 0.05). ND, non-detected; NA, not analyzed because the fungus was unable to grow in this condition.

Figure 2 shows the OTA concentration produced by *A. carbonarius* and *A. niger* as determined by HPLC, and the AFB_1_ levels produced by *A. flavus*, as measured by ELISA. The mycotoxin concentration was not determined under the conditions in which the fungi could not grow.

The results (Figure 2a) indicated that the isolates *Rhodococcus* 8-G8 and *Bacillus* 7-B7, which produced the highest reduction in *A. carbonarius* growth, were also able to significantly decrease the OTA concentration in the co-cultivation plates below the detection limit.

Something similar occurred in the case of *A. niger* (Figure 2b). In these plates, *Bacillus mycoides* 7-B7 and *Arthrobacter* sp. 7-B8 reduced fungal growth and decreased OTA concentration, also reaching values below the detection limit. Despite effectively reducing *A. niger* growth, the presence of *Mycetocola* 3-C10 in CYA plates significantly increased the OTA concentration with respect to control.

The results regarding AFB_1_ produced by *A. flavus* (Figure 2c) indicated that five potential BCAs significantly reduced toxin concentration in CYA plates in all *A. flavus* strains below the detection limit. The presence of isolates of the genus *Arthrobacter* (6-F11, 7-B8), *Rhodococcus* 8-G8, and *Bacillus mycoides* 7-B7 decreased mycotoxin concentration with reduction near 99% in all cases. On the other hand, *Mycetocola* 3-C10 significantly reduced the AFB_1_ concentration with respect to control in the co-cultures with one of the *A. flavus* isolates.

### 2.4. Viability of Potential BCAs after Lyophilization

**Figure 3:**
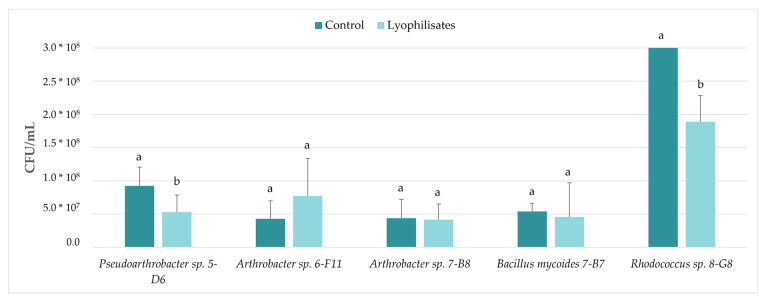
Effect of lyophilization on the viability of isolates. The bars indicate the mean of the three biological replicates and the thin lines their standard deviation values. The letters above each of the bars indicate statistically different groups generated in the Student’s *t* test.

The pre- and post-lyophilization counts are shown in Figure 3. The isolate 3-C10 was discarded because it did not show promising results in previous studies. Only in two isolates did lyophilization significantly reduce their viability: *Pseudoarthrobacter* sp. 5-D6 (*p* = 0.045) and *Rhodococcus* sp. 8-G8 (*p* = 0.000).

## 3. Discussion

Because mycotoxins in grapes present significant problems for food safety and the economy [4,6], it is essential that effective mycotoxin control be established to prevent their accumulation in the field and post-harvest since there are no sufficiently safe and efficient detoxification methods for their removal from foodstuffs. In fact, the European Union specifically forbade chemical treatments to aid in the detoxification of this compound type [1]. In this sense, the use of microorganisms as a BCA in vineyards is one of the most promising strategies for controlling the growth and production of toxins, thus reducing the need for harmful chemicals. However, the development of BCAs for the control of toxin-producing fungi may require a slightly different approach because many toxigenic species under environmental stress may not be able to grow and colonize the host effectively yet still increase their ability to produce mycotoxins. In our work, we have described this phenomenon in the case of *Mycetocola* sp. 3-C10 which increased OTA production by *A. niger* despite having significantly reduced fungal growth. Therefore, this finding supports the relevance of focusing on both fungal growth and mycotoxin production in the development of new BCAs for the control of mycotoxigenic fungi [21].

The main characteristics of an ideal biological control agent are genetic stability, efficacy at low concentrations, survival under adverse environmental conditions, growth on cheap substrates in fermenters, absence of pathogenicity to the host plant, non-production of potentially toxic metabolites to humans, resistance to the most commonly used pesticides, and compatibility with other chemical and physical treatments [22]. Moreover, since most plant pathogenic and toxigenic fungi originate in the soil, using the microbiota in untreated fields or organic crops that are in contact with them, is a good biocontrol strategy. In fact, the soil microbial community of bacteria, fungi, archaea, protists, and viruses represents the largest reservoir of biological diversity in the world, and might be a perfect place for finding microbial antagonists. Although previous studies on grapes have been conducted [23], the present work is the first to attempt to control fungi from their inocula in vineyard soils. It is well known that soils are the main source of toxigenic fungal contamination in vineyards [20], and their control in this environment might also reduce their presence in grapes and consequent mycotoxin contamination. Because of this, a sequential screening of microorganisms isolated from organic vineyard soils was carried out to search for potential BCAs against toxigenic fungi. This process facilitated the management of numerous microorganisms as potential antagonists and discarded many of them if they did not fit the characteristics of an ideal BCA [24].

First of all, it is essential in BCA development to avoid potential risks to people; therefore, the initial criterion has to be the absence of growth at 37 °C, the temperature at which human pathogens are able to grow. In this work, this first step reduced the number of BCA candidates by 93.2%, which allowed the subsequent steps to be applied to a much more manageable number of microorganisms. In addition, the potential BCAs selected in this study to be applied in vineyard soils, had to withstand environmental variations such as changes in humidity, pH, or temperature [24]. Therefore, the next screening steps were focused on their ability to survive under these extreme conditions. According to the results, most of the isolated microorganisms grew at pH 8 and 4 °C, possibly because they had been isolated from agricultural areas and subjected to a wide range of temperatures and alkaline soils. On the other hand, few isolates of this study grew at water activity (a_w_) 0.93. Whether they would not survive in low water activity is uncertain. Soil is a constantly changing ecosystem, and the microorganism might remain dormant under conditions of water stress waiting for optimal conditions for their development. The steps performed in the screening are inexpensive and simple to carry out, and after the complete process, 96.9% of the microorganisms tested were discarded, which showed sequential screening as a useful tool for selecting potential BCAs.

Eventually, only the most promising microorganisms that fulfilled all the criteria for commercial success as a potential BCA were identified. In the case of vineyards, biological control using yeasts and lactic acid bacteria was considered to be an environmentally friendly alternative to chemical treatments for controlling the growth and sporulation of OTA-producing fungi, such as *A. niger* and *A. carbonarius*, in grapes [23]. However, in our work, the four yeast isolates were discarded since they were classified as *Cryptococcus* sp., and this genus comprises several pathogenic species. Regarding the results of the bacteria and specifically actinobacteria, four genera were identified, including *Arthrobacter* and *Rhodococcus*, which have been extensively studied as BCAs and for their bioremediation potential as discussed below.

Actinobacteria are Gram-positive filamentous bacteria found in many ecological niches including soil. They present an interesting natural and cost-effective alternative for the efficient biodegradation of mycotoxins [25]. In fact, these bacteria have been widely investigated for their ability to produce molecules of interest such as antifungal compounds and enzymes that produce strong antagonistic capacities against fungi [26]. On the other hand, outside the group of actinobacteria, *Bacillus mycoides* was identified among the potential BCAs selected. Most *Bacillus* species are saprophytes that are able to use the great diversity of organic soil substrates to develop high genetic and functional diversity [27].

The results of the confrontation between the microorganisms and the selected fungi together with the analysis of the mycotoxins they produced, indicated that the most promising strain (isolate 7-B8) belonged to the genus *Arthrobacter*. Its species are very resistant to desiccation and nutrient deficiency, and although they have been isolated from humans and other animals, they are considered rare in clinical samples [28]. The isolates of this genus are, therefore, of interest for study as potential BCAs; in fact, *Arthrobacter* sp. strains such as FP15 have been found to secrete compounds that efficiently inhibit *A. carbonarius* growth [29]. Other species are known to secrete chitinase and antibiotic compounds with inhibitory effects on a wide range of plant pathogenic or xylophagous fungi [30,31,32]. In addition, the potential of these rhizobacteria in wild *Lauraceae* against *Fusarium solani* and *F. oxysporum* has been described [33]. It is worth noting the differences among the different isolates of this genus as observed in the cases of 7-B8 and 6-F11 which present a completely different behavior following confrontation with the mycotoxigenic fungi. Therefore, the screening allowed us to reduce the number of microorganisms so that we could study specific characteristics at the isolate and strain levels and at the not-so-specific genus and species level. Furthermore, the results of freeze-drying using 7-B8 were very promising for the commercialization of this bacterium as a potential BCA since there was no reduction in viability.

On the other hand, the second-best performing microorganism was *Bacillus mycoides* 7-B7, as mentioned above. Other studies already isolated *Bacillus* species from the rhizosphere of vineyards to function as a biocontrol agent against ochratoxigenic strains of *A. ochraceus* and *A. carbonarius* on grapes [34], and from the tomato rhizosphere to work against other toxin-producing fungi such as *F. oxysporum* [35]. In addition, these species are considered biological control agents against phytopathogenic species such as *Alternaria* [36]. *Bacillus amyloliquefaciens* B4 and *Bacillus cereus* species [37,38] have been reported to be highly effective against plant pathogenic fungi that cause post-harvest diseases. In particular, the species *Bacillus mycoides* has been described as a potential biocontrol agent against plant pathogenic fungi such as *Cercospora beticola* Sacc. [39]. There are currently several commercial formulations, such as Fungisei (Seipasa), Serifel (BASF), Taegro (Syngenta), and LifeGard WG (Certis), that include strains of the *Bacillus* genus as an active ingredient due to their colonizing capacity, easy reproduction and high persistence associated with their capacity to form endospores. This feature is of special interest as it allows them to survive under abiotic stress conditions, which facilitates production and storage for long periods of time [40]. Furthermore, these data are in agreement with the results obtained for this isolate after a freeze-drying assay, making it a good candidate for commercialization as a potential BCA.

Finally, the third-best performing microorganism (isolate 8-G8) belonged to the genus *Rhodococcus*. Species belonging to this genus contain large genomes that allow them to have different versatile catabolic pathways, giving them, in turn, the ability to uptake and metabolize hydrophobic compounds. In addition, many *Rhodococcus* species form biofilms and persist in adverse conditions [41]. This characteristic indicates that this genus might be explored with regard to the degradation of mycotoxins; in fact, *Rhodococcus erythropolis* ATCC 4277 is known to degrade AFB_1_ [42]. In this case, a reduction in viability was observed after the freeze-drying trial, so the following studies may focus on improving viability for commercialization.

Lastly, although none of the bacteria genera that showed the best results as a potential BCA are currently on the EFSA QPS (Qualified presumption of safety) list [43], they are good candidates for inclusion since they have not been described as posing a health risk or danger to the environment. In fact, some isolates of the genera that we have obtained as potential BCA are already included in the list of GRAS (Generally recognized as safe) organisms prepared by the FDA; therefore, in the future, it might not be difficult to include them in the QPS list elaborated by EFSA. The subsequent studies will aim at their full characterization including a complete identification at the species level and an investigation of the mechanisms under their biocontrol ability. Moreover, it is essential to demonstrate their in vivo biocontrol potential in soils. All the subsequent studies will be performed in collaboration with a specialized company in the development of biopesticides to ensure that these organisms comply with the current legislation.

## 4. Conclusions

In view of the above information, it is necessary to emphasize that sequential screening presents a good strategy for selecting potential BCAs for mycotoxin control in vineyards. *Arthrobacter* sp. isolate 7-B8 and *Bacillus mycoides* 7-B7 significantly reduced in vitro growth of all *A. carbonarius*, *A. niger* and *A. flavus* strains tested as well as their ability to produce mycotoxins. On the other hand, it is clear that the development of new BCAs towards mycotoxigenic fungi must not be based solely on the evaluation of their effect on fungal growth. In this work, we found that *Mycetocola* sp. 3-C10 significantly reduced the development of the three fungal species but its presence supposed an increase in OTA production by *A. niger*.

## 5. Materials and Methods

### 5.1. Sample Collection and Processing

Soils from eight organic vineyard fields were sampled from different regions of Spain: Madrid (samples 3, 4 and 9); Valencia (sample 5); Burgos (samples 6, 7, and 8) and Toledo (sample 10) during September, October, and November 2019. Sampling was conducted by parceling each field equally into three transects, the length of which was selected as the distance covered while we walked making four random and equidistant stops. In each transect, 300 mL of subsurface soil volume was collected, under the most aseptic conditions possible, to obtain a soil sample per field of 3.6 L.

Each sample was processed independently. First, all the subsamples were mixed and left to dry at room temperature on filter paper for 48–96 h. Then, the samples were passed through a 200 µm pore size sieve and subsamples of approximately 50 g were stored at 4 °C for later use.

### 5.2. Isolation of Soil Microorganisms

One gram of each soil sample was used for microorganism isolation using the serial dilution technique: decimal dilutions (up to 10^−5^) in sterile saline solution. Then, the dilutions were cultured in duplicate on Rose Bengal Agar plates with Chloramphenicol (Pronadisa, Madrid, Spain), 10% Trypticasein Soy Agar (TSA) (Pronadisa, Madrid, Spain) and a specific medium for actinobacteria, Actinomycete Isolation Agar (Sigma-Aldrich, Darmstadt, Germany) to discriminate among yeast, total bacteria and actinobacteria. The plates were incubated at 25 °C for 48 h.

Subsequently, a maximum of 30 colonies of bacteria, actinobacteria and yeasts were selected for each sample to continue with screening (total 513 colonies). Colonies were selected based on apparently distinct morphological characteristics and appearance. Those selected were re-isolated by streaking in TSA or PDA plates, as appropriate. The plates were incubated at 25 °C for 48 h and, finally, the isolated microorganisms were stored as cell suspensions at –80 °C in 15% glycerol.

### 5.3. Initial Screening of Microorganisms for Absence of Growth at 37 °C

This test was performed in 96-well plates containing 250 μL of Trypticasein Soy broth (TSB) (Pronadisa, Madrid, Spain) or Potato Dextrose broth (PDB) (Pronadisa, Madrid, Spain), depending on the type of inoculum (bacteria or actinobacteria). Then, the 240 bacterial colonies from the stock TSA plates were seeded into the wells with TSB and the 240 actinobacterial colonies from specific medium for actinobacteria plates were seeded into the wells with PDB. Some wells were left uninoculated as blanks. The 33 yeast isolates selected were cultured on PDA plates and incubated for 3 days at 37 °C. All microorganisms that did not show growth were selected for subsequent analysis and their potential as BCAs was tested.

### 5.4. Sequential Selection of Soil-Isolated Microorganisms

Sequential screening was carried out following the recommendations of Köhl et al. [24]. Considering the absence of growth at 37 °C as a criterion for discarding potential pathogens, a reconfirmation of the inability to grow at this temperature was determined by a longer incubation. Cellular suspensions of the previously selected microorganisms were made in sterile 0.9% *w*/*v* saline up to the 0.5 McFarland turbidity standard. In total, 200 microliter volumes of each bacterial, actinobacterial or yeast suspension were inoculated into 100 mL flasks with 20 mL of TSB or PDB (as appropriate). The flasks were incubated at 37 °C without shaking for 14 days, and only microorganisms that did not show any growth were selected.

Afterwards, the ability of the microorganisms to grow and survive at different environmental conditions was tested. Cellular suspensions were made as described above. Then, the experiment was performed in three 96-well plates testing different parameters including temperature (4 °C), pH (5 and 8) and water activity (a_w_ 0.93). For this purpose, TSB and PDB media were modified to reach the correct pH or a_w_ level, and 25 μL of each microorganism suspension was inoculated in 225 μL of medium. Three replicates of each sample were performed. Plates containing the media with the different pH and a_w_ 0.93 were incubated at 25 °C for 14 days. A fourth plate with the common PDB or TSB medium was incubated at 4 °C for 14 days as control.

Finally, after incubation, the growth of the microorganisms was checked as indicated in Section 2.4, and those that had grown under the four conditions were selected.

### 5.5. Identification of Microorganisms

The bacteria and yeasts that passed all the screening processes were identified by sequencing a partial region of the 16S rDNA and the ITS1-5.8S-ITS2 region, respectively. Firstly, colony PCR was performed to amplify these regions using the primer pairs Y1/Y2 [44] and ITS1/ITS4 [45] and the amplification programs designed by Delgado et al. [44] and Henry et al. [46], respectively, modified by adding an initial denaturation of 10 min to lyse the cells. PCR reactions were carried out on an Eppendorf Mastercycler Nexus^®^ thermal cycler (Eppendorf, Hamburg, Germany). All PCR products were visualized by horizontal electrophoresis on 1.5% agarose gels (Pronadisa, Madrid, Spain) in TAE buffer (40 mM Tris-Acetate, 1 mM EDTA) with 5 μL of Green Safe Premium (1 μg/mL) (NZYTech, Lisbon, Portugal). Electrophoresis was carried out in TAE buffer at 80 V for 25 min and visualized under UV light. The molecular weight marker used was NZYDNA Ladder V (NZYTech, Lisbon, Portugal). PCR products were purified using the NZYGelPure kit (NZYTech, Lisbon, Portugal) following the manufacturer’s protocol and their concentration was determined with a NanoDrop^®^ ND-1000 spectrophotometer (NanoDrop Technologies, Wilmington, DE, USA). Finally, they were sequenced at Macrogen Facilities (Spain). All PCR products were sequenced in both directions and the sequences obtained were aligned and processed using the UGENE^®^ program (UniPro, Surgut, Russia). Finally, their degree of similarity with other sequences deposited on databases was checked using the BLAST^®^ tool of NCBI (USA) (https://blast.ncbi.nlm.nih.gov/Blast.cgi (accessed on 26 September 2022). All of them were identified with a percentage of identity greater than 98%.

### 5.6. Biocontrol Assay Using the Selected Microorganisms against Toxin-Producing Fungi

In total, three aflatoxin-producing strains of *A. flavus* (19.1.1, 19.4.1 and 19.7.1), OTA-producing strains of *A. carbonarius* (273, 282 and 350) and *A. niger* (258 and 19.1.2) all isolated from grapes, and *A. niger* AV.10 isolated from oats were selected to test the potential of the eight selected bacteria to reduce in vitro fungal growth. Fungal strains were cultured on PDA and incubated at 28 °C for five days. Subsequently, spore suspensions were prepared in sterile saline solution, and the concentration was determined using a Thoma counting chamber (Marienfeld, Lauda-Königshofen, Germany) and adjusted to a final concentration of 10^6^ spores/mL. The bacteria were cultured in PDA and TSA plates for 48 h at 25 °C and a cell suspension was prepared to obtain the 0.5 McFarland turbidity standard.

The effect of bacteria on fungal growth was evaluated in CYA medium (45.5 g/L of modified Czapek–Dox agar (Pronadisa, Madrid, Spain) and 5 g/L of yeast extract (Pronadisa, Spain)). The potential BCAs were added to the medium and the same amount of saline solution was included in the control plates instead of bacteria. All plates were inoculated with 2 μL of each fungal spore suspension (3 mm diameter drops) in the center of the plate. The fungal colony diameter was measured daily in two directions until the surface of the control plates was fully colonized, which took 5 days for *A. carbonarius*, 7 days for *A. flavus*, and 6 days for *A. niger*. Growth rate (mm/day) was calculated from a linear model by plotting diameter (mm) against time (day) both for control plates and bacterial-supplemented ones.

AFB_1_ and OTA concentrations in the CYA plates were determined to evaluate the effect of potential BCA on the corresponding toxigenic fungi. For this purpose, three agar plugs were extracted from the border, middle and center of the fungal growth zone using a 5 mm diameter punch. The disks were placed into 1.5 mL Eppendorf tubes and 1 mL of methanol was added. The toxins were extracted by vortexing for 20 min. The methanolic extracts were filtered through 0.45 μm pore-sized filters (Fisherbrand, Madrid, Spain).

### 5.7. Study of the Reduction Capacity of Ochratoxin A and Aflatoxin B1 by Selected Bacteria as Possible BCA

Quantification of AFB_1_ was performed using the RIDASCREEN^®^ Aflatoxin B1 30/15 Art. No. R1211 (R-Biopharm, Darmstadt, Germany), following the manufacturer’s instructions. The kit is based on a competitive ELISA assay, in which the absorbance measured is inversely proportional to the mycotoxin concentration of the sample. The resulting color reaction was measured at 450 nm with a plate reader (Dutscher, Bernolsheim, France).

A six-point standard curve was prepared using samples with different concentrations of AFB_1_ (0; 1; 5; 10; 20; 50 μg/L) (%(B/B_0_) = −35.291 log (concentration) + 76.952; R^2^ = 0.988) included in each RIDASCREEN^®^ ELISA kit. The percentage of absorbance (% (B/B_0_)) was calculated by the following formula: % (B/B_0_) = (Standard absorbance of sample (B)/Absorbance of blank (B_0_)) × 100.

The absorbance data obtained in the detoxification test were interpolated from the standard curve to obtain the concentration value of AFB_1_ present in the samples.

OTA concentration of the extracts was measured by HPLC. A standard of OTA was dissolved in methanol at a concentration of 5.0 mg/mL and stored at 4 °C in a sealed vial until use. The concentration in the stock solution was checked by UV spectroscopy according to AOAC Official methods of analysis, chapter 49 [47] Working standard solutions (0.5, 0.01, 0.005, 0.001 and 0.0005 mg/ mL) were prepared by appropriate dilution of known volumes of the stock solution with the HPLC mobile phase and used to obtain calibration curves in the appropriated chromatographic system. OTA was determined by an Agilent Technologies 1260 Infinity HPLC system with quaternary pump (G1311B) coupled to a fluorescence detector (G1321B), using an analytical column Kinetex PFP 100 Å 5 μm, 4.6 × 150 mm (Phenomenex^®^, Torrance, CA, USA). Excitation and emission wavelengths were set, respectively, at 330 and 463 nm. Mobile phase consisted of acetonitrile (A), methanol (B) and acetic acid (C) 0.1% in the following gradients:

Time 0 min: 15% A, 0% B, 85% C;

Time 5 min: 14% A, 27% B, 59% C;

Time 12 min: 90% A, 0% B, 10% C; and

Time 15 min: 15% A, 0% B, 85% C rebalance column.

The mobile phase flow rate was 1 mL/min. The injection volume was 100 µL. The retention time was 11.8 min. The limit of detection in the extract was 0.001 µg/mL and the limit of quantification was 0.003 µg/mL.

### 5.8. Viability of potential BCAs after Lyophilization

The bacteria that yielded better results as potential BCAs against toxigenic fungi were cultured in flasks with 15 mL of PDB or TSB, depending on whether they were actinobacteria or bacteria, and incubated for 48 h at 25 °C. Subsequently, 5 mL of these cultures were concentrated by centrifugation of the sample and following removal of the supernatant, carrying out 2 biological replicates of each microorganism. This biomass was mixed with 125 μL of sterile 20% Sveltesse^®^ skim milk (Nestlé, Barcelona, Spain) and 125 μL of PDB or TSB in lyophilization tubes. After homogenization, the lyophilization tubes were frozen for 2 h at –20 °C and then at –80 °C for a minimum of 4 h. Finally, these tubes were lyophilized in a Cryodos lyophilizer (Telstar, Barcelona, Spain) for 24 h. The lyophiles were stored at 4 °C until needed. The biomass of the third Eppendorf was resuspended in 1 mL of saline to be used as a preliminary control.

To check the loss of viability from freeze-drying, viable counts (CFU/mL) were carried out before and after. For this purpose, a bank of decimal dilutions in saline solution and subsequent colony count in PDA and TSA plates, as appropriate, was performed, making three replicates for each experimental condition and replicate. The plates were incubated at 25 °C for 72 h. Loss of viability was obtained from the difference in CFU/mL pre-lyophilization.

### 5.9. Statistical Analysis

Statistical analyses were performed using the StatGraphics Centurion XVII V.17.2.04 software (Statpoint Technologies Inc., Warrenton, VA, USA). The normality and homoscedasticity of the data were tested by the Shapiro–Wilk and Bartlett tests. When the data did not fit to normality and homoscedasticity, they were either log-transformed or x^3^–transformed.

Afterwards, an analysis of variance (ANOVA) was conducted followed by Fisher’s LSD post hoc test for checking differences among group means. In all cases, the significance level was set at *p* < 0.05.

## Figures and Tables

**Table 1:** Growth of soil bacteria isolated from actinomycete isolation agar (AB), bacteria isolated from TSA (BC) and yeasts isolated from PDA (Y) at different growth conditions: 4 °C, pH 5, pH 8 and a_w_ 0.93. Thecells with a cross indicate growth in each of the conditions. The bold isolate name indicates its selection in subsequent studies because it met all established criteria. All isolates and conditions were tested in triplicate.

	GROWTH CONDITIONS					GROWTH CONDITIONS					GROWTH CONDITIONS			
Isolated Microorganism	4 °C	pH5	pH8	a_w_ 0.93	Isolated Microorganism	4 °C	pH5	pH8	a_w_ 0.93	Isolated Microorganism	4 °C	pH5	pH8	a_w_ 0.93
**AB 3-C10**	x	x	x	x	AB 7-B9			x	x	AB 8-F3		x	x	
AB 5-D2	x		x	x	**AB 7-B10**	**x**	**x**	**x**	**x**	**AB 8-F7**	x	x	x	x
**AB 5-D6**	x	x	x	x	**AB 7-C6**	**x**	**x**	**x**	**x**	**AB 8-G8**	x	x	x	x
AB 6-E4			x		AB 7-C8			x	x	AB 8-E6		x	x	x
AB 6-E9	x		x	x	**AB 7-C10**	x	x	x	x	**AB 8-E10**	x	x	x	x
AB 6-F4	x		x	x	AB 7-D4	x		x		**AB 10-G7**	x	x	x	x
**AB 6-F9**	x	x	x	x	AB 7-D6	x			x	BC 3-D6	x		x	x
**AB 6-F11**	x	x	x	x	AB 7-D7	x	x	x		**BC 7-B7**	x	x	x	x
AB 6-G10	x		x		**AB 7-D8**	x	x	x	x	**Y 6-6**	x	x	x	x
AB 7-B6	x	x	x		AB 7-D11			x	x	**Y 6-7**	x	x	x	x
**AB 7-B8**	x	x	x	x	AB 8-G4	x		x	x	**Y 9-9**	x	x	x	x
										**Y 5-3**	x	x	x	x

## Data Availability

Raw data are available upon request, please contact the corresponding author.
